# *AKT2* Exon 3 Variants and Their Prevalence in Prostate Cancer Patients: Insights from a Jordanian Clinical Cohort

**DOI:** 10.3390/ijms27104228

**Published:** 2026-05-09

**Authors:** Nuseibah Rahahlah, Zina Al-Alami, Mohammed S. Alorjani, Asmaa Al-Smadi, Sewar Obeidat, Raed Otoom, Raed M. Al-Zoubi, Samir Al Bashir, Mazhar Salim Al Zoubi

**Affiliations:** 1 Department of Medical Laboratory Sciences, Faculty of Allied Medical Sciences, Al-Ahliyya Amman University, P.O. Box 115, Amman 19111, Jordan; rnosiba@gmail.com; 2 Department of Pathology and Microbiology, Faculty of Medicine, Jordan University of Science and Technology, Irbid 22110, Jordan; msalorjani@just.edu.jo (M.S.A.); smalbashir9@just.edu.jo (S.A.B.); 3 Department of Basic Medical Sciences, Faculty of Medicine, Yarmouk University, Irbid 21163, Jordan; asmaasmadi@gmail.com (A.A.-S.); obeidatsewar3@gmail.com (S.O.); raedotoom10@gmail.com (R.O.); mszoubi@yu.edu.jo (M.S.A.Z.); 4 Surgical Research Section, Department of Surgery, Hamad Medical Corporation, Doha P.O. Box 3050, Qatar; ralzoubi@hamad.qa; 5 Department of Biomedical Sciences, QU-Health, College of Health Sciences, Qatar University, Doha 2713, Qatar; 6 Department of Chemistry, Jordan University of Science and Technology, P.O. Box 3030, Irbid 22110, Jordan

**Keywords:** adenocarcinoma, *AKT2*, kinase, mutations, prostate cancer, good health and wellbeing

## Abstract

The *AKT2* gene, located on chromosome 19, encodes a protein involved in key cellular processes like metabolism, proliferation, and survival. Abnormalities in the PI3K/AKT pathway, including *AKT2*, contribute to tumor progression. AKT2 promotes cell survival, growth, and resistance to therapy. Overexpression or hyperactivation of AKT2 is linked to prostate cancer (PC) development, making it a potential therapeutic target. This study aimed to investigate the frequency and distribution of *AKT2* variants in a cohort of Jordanian men diagnosed with PC and to evaluate the relationship between these genetic variations and clinicopathological parameters, including age, tumor stage, PSA levels, and Gleason score. Formalin-fixed paraffin-embedded (FFPE) tissue samples (*n* = 123) were collected from Jordanian patients diagnosed with prostate adenocarcinoma. The collected samples underwent DNA extraction, followed by PCR amplification. Subsequently, exon 3 of *AKT2* was sequenced. The prevalence of *AKT2* mutations was 5.7% in the population studied. Six mutations were identified: two missense mutations (Pro51Ser and Gly33Ser), two synonymous, one splice acceptor, and one intron variant. The variants were not significantly correlated with clinical parameters; however, the prevalence of the *AKT2* mutations suggests potential relevance to PC pathogenesis. The prevalence of *AKT2* mutations in the current cohort suggested a potential role of *AKT2* in PC pathogenesis in the Jordanian population. Further genetic studies covering the whole *AKT2* gene and the downstream pathway are required for a better understanding of PC genetics.

## 1. Introduction

Prostate cancer (PC) is the fourth most frequent type of cancer diagnosed in 2022 worldwide. In the USA, PC is the second most prevalent cancer-specific cause of death, representing 7.3% of all newly diagnosed cancers [1,2]. These statistics underscore the urgent need for effective diagnostic and therapeutic strategies [3]. The etiology of PC is unclear; however, several risk factors have been reported to be associated with the development of PC, including age, ethnicity, genetic factors, family history, environmental factors, diet, and lifestyle [4,5,6,7]. In addition, genetic predisposition is currently investigated to understand the molecular basis of PC [8,9]. Genetic analyses have identified multiple genes for hereditary prostate cancer, with mutations in DNA repair genes such as *BRCA1*, *BRCA2*, *TP53*, and *ATM* [10,11,12,13,14]. Other genes that have been correlated with PC include *HOX*, *MSR1*, *RNase L (HPC1)*, *ELAC2/HPC2*, and *AKT1* [15,16,17,18,19]. In addition, carcinogenesis might be a consequence of many genetic alterations, including point mutations, single-nucleotide polymorphisms (SNPs), and somatic copy number alterations [20,21].

AKT, also known as protein kinase B (PKB), is a serine-threonine protein kinase comprising three isoforms: AKT1/PKBα, AKT2/PKBβ, and AKT3/PKBγ [22]. It comprises three main regions: the N-terminal pleckstrin homology (PH) domain, the central catalytic domain, and the C-terminal regulatory region. The PH domain plays a crucial role in recruiting AKT to the cell membrane, whereas the central catalytic domain and C-terminal regulatory region are essential for activating AKT kinase. Approximately 3–5% of tumors exhibit mutations in the highly similar kinases, *AKT1*, *AKT2*, and *AKT3* [23].

Overexpression or activation of AKT is present in many cancers, such as ovarian, lung, and pancreatic cancers, and is known to be associated with the proliferation of cancer cells [24]. Recent research in Jordan by Alasmar et al. in 2024 reported genetic alterations in the AKT1 PH domain, including exons 3 and 4, and a high frequency of *AKT1* mutation in PC patients in Jordan, with two novel missense mutations in the PH domain [15].

AKT2, known as RAC-beta serine/threonine-protein kinase (PKBβ), is particularly important in glucose metabolism and insulin signaling pathways. A lack of it is associated with insulin resistance and it is widely expressed in insulin target tissues, such as the liver, skeletal muscles, and adipose tissue [25]. Changes in AKT2 or a disturbance of its function is likely to result in metabolic disorders, including insulin intolerance and type II diabetes [26]. Furthermore, AKT2 is involved in tumorigenesis and the metastatic process. Moreover, activation of AKT2 results in enhanced invasiveness and migration in ovarian and breast carcinomas [27].

In a previous report, we identified novel pathogenic missense mutations in exon 3 of the closely related *AKT1* gene in Jordanian PC patients, establishing a precedent for exon 3 as a mutation-bearing region in the AKT family within this population. In addition, the rationale for focusing specifically on exon 3 of *AKT2* stems from the functional importance of the region it encodes. Exon 3 spans part of the N-terminal pleckstrin homology (PH) domain, which mediates membrane recruitment of AKT2 via binding to PIP3—a critical step in kinase activation. This study aims to investigate the frequency and distribution of exon 3 *AKT2* gene variants in a cohort of Jordanian men with PC and to investigate the relationship between these genetic variations and clinicopathological parameters, including age, tumor stage, prostate-specific antigen (PSA) levels, and Gleason score. We acknowledge, however, that investigation of additional exons—particularly those encoding the kinase domain and the C-terminal regulatory region—will be necessary for a comprehensive genetic portrait of AKT2 in PC, and this is outlined as a priority for future work.

## 2. Results

A total of 123 samples were collected from PC patients, with ages ranging from 39 to 96 years, reflecting a broad age distribution. The study focused on the clinicopathologic parameters associated with these patients. Table 1 presents these parameters comprehensively, including the number of samples, mean age, mean PSA level and median Gleason score.

**Figure 1:**
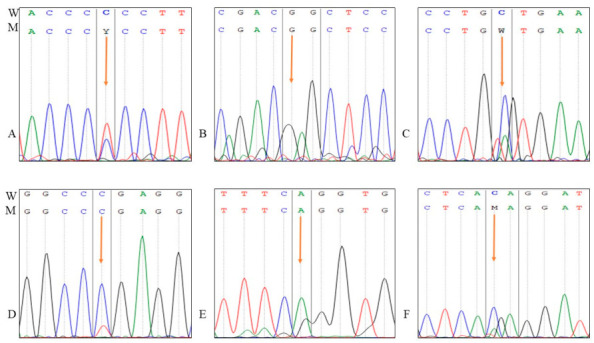
Sequence chromatograms of recorded variants in *AKT2* gene exon 3: (**A**): rs201145928, (**B**): rs1975585202, (**C**): rs1975586566, (**D**): rs1347826243, (**E**): rs1975588070, and (**F**): rs2145305645 (W: wild genotype and M: mutant genotype). The arrows point to the mutations. G in black: Guanine, A in green is Adenine, C in Blue is cytosine and T in red: Thymine.

All samples were screened for the mutations in exon 3 of the *AKT2* gene and recorded six different mutations in seven samples: two missense variants (rs201145928 (C>T, Pro51Ser), rs1975585202 (C>T, Gly33Ser)), two synonymous variants (rs1975586566, and rs1347826243), one splice acceptor variant rs1975588070, and one intron variant rs2145305645. The mutations detected in the exon 3 *AKT2* gene are presented in Table 2 and Figure 1. The two missense variants—rs201145928 (Pro51Ser) and rs1975585202 (Gly33Ser)—are rare variants with minor allele frequencies well below 0.1% in gnomAD, confirming their rarity in the general population. The two synonymous variants (rs1975586566 and rs1347826243) have similarly low allele frequencies. The splice acceptor variant (rs1975588070) and the intron variant (rs2145305645) are also rare. None of the six variants is flagged as a benign common polymorphism.

COSMIC (Catalogue of Somatic Mutations in Cancer) and cBioPortal were searched for prior reports of each identified variant across all cancer types. The search revealed that rs201145928 (Pro51Ser) and rs1975585202 (Gly33Ser) have not been previously reported as somatic mutations in COSMIC or in any cBioPortal cancer dataset, supporting their novelty. The remaining variants (synonymous, splice acceptor, and intron) are similarly absent from COSMIC somatic mutation records.

The association between wild-type samples and mutant samples based on clinical and pathologic characteristics (age, PSA level, and Gleason score) was evaluated using the Chi-square test Table 3. There is no statistical significance (*p* value > 0.05), and the percentage of mutant samples in prostate samples and the percentage of mutation of exon 3 (*n* = 123) was 5.7%.

**Figure 2:**
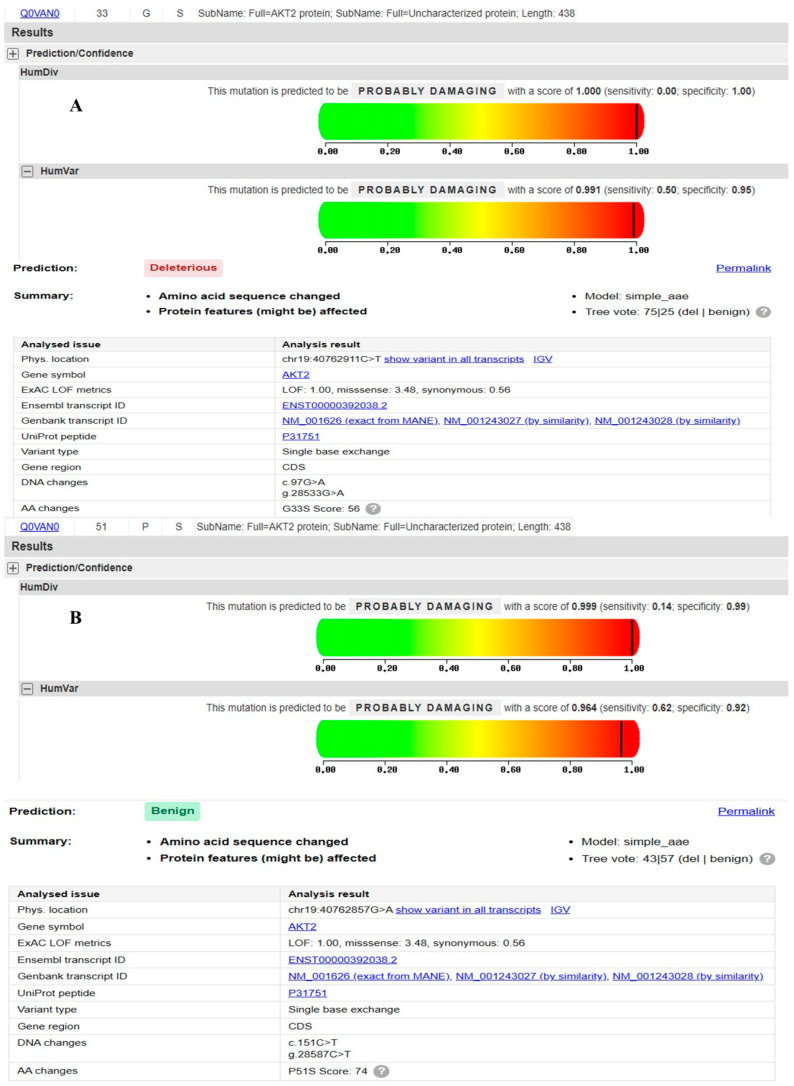
PolyPhen-2 and MutationTaster predictions for (**A**) rs1975585202(G33S) and (**B**) rs201145928 (P51S).

The functional impact of two missense variants located in exon 3 of the *AKT2* gene, rs1975585202(G33S) and rs201145928 (P51S), was evaluated using two in silico prediction tools: PolyPhen-2 (http://genetics.bwh.harvard.edu/pph2/, accessed on 6 December 2025) and MutationTaster (https://www.genecascade.org/MutationTaster2021/, accessed on 6 December 2025). The glycine to serine substitution at position 33 demonstrated strong concordance (agreement) between both tools, suggesting a high probability of functional disruption, Probably Damaging and deleterious, as shown in Figure 2A. The analysis for the proline to serine substitution at position 51 showed conflicting results between the two platforms: PolyPhen-2 predicted the change to be Probably Damaging, and MutationTaster classified the variant as Benign (Figure 2B).

**Figure 3:**
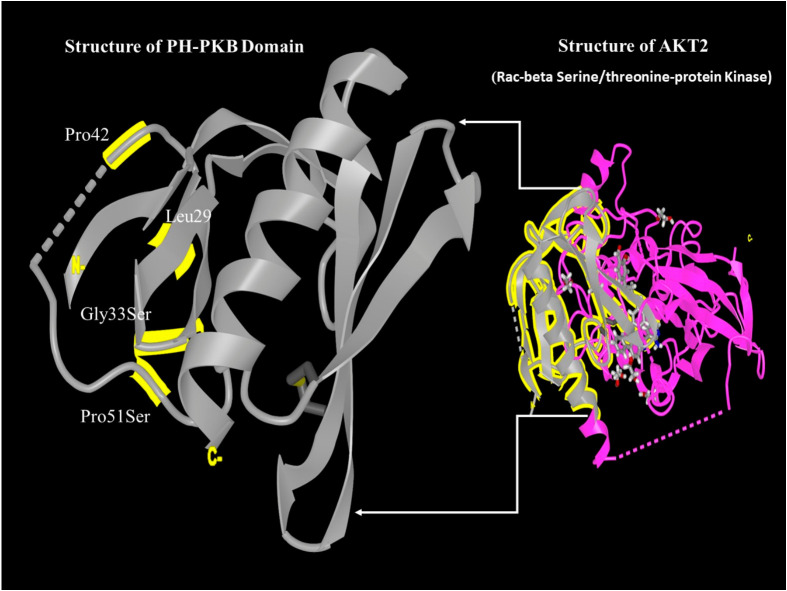
Structural modeling of the AKT2 protein and its PH-PKB domain (labeled with residue name and number highlighted in yellow). The dashed line indicates specific inter-atomic distances/interactions.

The tertiary structure of the AKT2 protein was modeled to visualize the identified substitutions using the NCBI structure database (https://www.ncbi.nlm.nih.gov/Structure/pdb/9C1W, accessed on 30 July 2025). This structural mapping focuses on the PH-PKB domain as shown in (Figure 3).

## 3. Discussion

Prostate cancer (PC) is a genetically diverse disease characterized by the accumulation of germline and somatic mutations that affect critical oncogenic and tumor suppressor pathways [28]. In the current study, two missense variants, two synonymous variants, one splice acceptor variant, and one intron variant were detected, and the mutation frequency of exon 3 in the *AKT2* gene was found to be 5.7% of the tested samples.

The current results showed that mutation ns in this area do exist in a proportion of patients with PC, while they might play only a minor molecular role. This result also supports that *AKT2* exon 3 variants could be a cause of the molecular diversity of PC, although this is a relatively small proportion. Compared with the current literature in other populations, the current result may signify the genetic background of the population or the sequencing methods. Prior studies have reported *AKT2* variants in various disease contexts, including blood cancer in a Pakistani population [29], noise-induced hearing loss sensitivity in a Chinese cohort [30], fasting insulin levels and type 2 diabetes risk in a Finnish population and a Chinese Han population [31,32], and a small percentage of patients from Japan with lung cancer [33].

Based on the current data, no significant correlation between mutation status and major clinicopathological variables such as age, PSA, and Gleason score was detected. Therefore, the detected variants do not appear to affect the traditional clinicopathological variables of PC. The same result was detected when ERG expression appeared independent of Gleason score, age, and PSA levels in patients with PC in Southwestern Uganda [34]. However, it should be considered that a lack of statistical significance does not essentially mean that there is no biological effect; the limited sample size (*n* = 123) might have contributed to this non-significance.

The Gly33Ser (G33S) variant is predicted to be damaging by PolyPhen-2 and MutationTaster. Glycine 33 is located in the PH domain loop region involved in PIP3 binding; a serine substitution at this position introduces a bulkier, polar side chain that is predicted to disrupt PIP3 interaction, potentially impairing membrane recruitment and reducing AKT2 activation (loss-of-function mechanism). The Pro51Ser (P51S) variant yielded conflicting predictions (PolyPhen-2: Probably Damaging; MutationTaster: Benign), making functional inference less certain. In the context of PC, while canonical AKT2 overactivation is oncogenic, loss-of-function variants in the PH domain may paradoxically affect the PI3K/AKT/mTOR equilibrium in tumor-specific ways, potentially influencing metabolic reprogramming or resistance to androgen deprivation therapy. Functional assays—including kinase activity assays and membrane localization studies using cell lines transfected with mutant constructs—are required to definitively determine whether these variants confer a gain or loss of function, and we frame this as a primary goal of future experimental work.

The current results support a few reports about the role of *AKT2* in the pathogenesis of PC; activation of the PI3K-AKT-mTOR pathway by oncogenic factors is prevalent in prostate cancer and promotes the development and progression of PC [35]. Even in the absence of alliances with clinicopathological characteristics, biologically, *AKT2* alterations could play a role in the disruption of the PI3K/AKT signaling pathway [36]. Future studies must include protein activity analysis, larger genomic series, and functional studies to define the consequences of these alterations.

Different studies suggested the important role of the PIK3AKT-mTOR signaling pathway in the development of different cancers, including PC. PIK3AKT-mTOR activation is strongly associated with oncogenesis and contributes to anti-apoptotic activities [37]. Therefore, the current results appeared to be in agreement with the proposed role of AKTs in the development of PC; for instance, overexpression of *AKT2*/protein kinase Bbeta resulted in up-regulating beta1 integrins and also increased invasion and metastasis in human breast and human ovarian cancer cells [38]. In addition, *AKT2* knockdown showed an increase in tumor growth of ovarian cancer in a mouse model [39].

Different studies showed that AKT2 is overexpressed is different types of cancer, such as ovarian, pancreatic cancers, colorectal cancer, and liver cancer reviewed in [40,41]. AKT2 is also reported to be overexpressed in various human cancers, including breast cancer [42], lung cancer [43,44], colon cancer [45], ovarian cancer [46], and pancreatic cancers [47]. However, a few reports showed genetic alterations in the *AKT2* gene and are limited to the E17K hotspot. Therefore, the current study supports the possible role of other genetic alterations in the *AKT2* gene, which will clarify the genetic portrait of PC.

In summary, the current study exhibited that exon 3 variants of *AKT2* can be detected in prostate cancer patients within this Jordanian population and that two missense mutations may have functional significance. Nevertheless, these variants did not show a significant association with clinical factors. Hence, while the results support the use of molecular methods and prediction, they could not indicate a contributing role for *AKT2* mutations in the promotion of tumor aggressiveness, outcome, or therapy response.

## 4. Materials and Methods

### 4.1. Samples Collection

The Department of Pathology at King Abdullah University Hospital provided Formalin-Fixed Paraffin-Embedded (FFPE) tissue samples from 123 PC patients who underwent prostatectomy from January 2003 to December 2015. The average age of patients who were enrolled and had PC was 71.57 years, with ages ranging from 39 to 96 years.

### 4.2. DNA Extraction

DNA from tissue samples was extracted using the ZYMO Research Quick-DNA FFPE Miniprep kit (Zymo Research, Irvine, CA, USA) following the manufacturer’s instructions. Firstly, tissue sections (5 sections, 10 μm each) were deparaffinized three times with xylene at 55 °C for 5 min, followed by three washes with absolute ethanol. Tissues were then dried at 55 °C for 2–3 h or overnight at room temperature. Proteinase K buffer (50 µL) and digestion buffer (400 µL) were added to the dried tissue, mixed thoroughly, and incubated overnight at 55 °C with shaking. Subsequently, the mixture was heated at 90 °C for 30–60 min to inactivate the proteinase K enzyme. DNA purification was performed as per the kit protocol. All collected samples were stored at −20 °C until needed, and for long-term storage for further analysis, DNA samples were stored at −80 °C. Nanodrop measurements were used to assess DNA purity and concentration.

### 4.3. Primer Design for AKT2 Exon 3 Amplification Using PCR

The PCR amplification of the *AKT2* gene exon 3 was achieved by using specific primers designed using the Primer3 and Blast primer websites (Chromosome 19 NC_000019.10) according to the National Center for Biotechnology Information (https://www.ncbi.nlm.nih.gov/, accessed on 30 July 2025) and the Ensemble genome browser (https://asia.ensembl.org/index.html, accessed on 30 July 2025), as presented in Table 4.

The PCR was carried out in a 30 μL reaction mixture with DNA polymerase 2X Green Master Mix (Promega Corporation, Madison, WI, USA), containing a reaction buffer at pH 8.5; 3 mM MgCl_2_; 400 μM of each dATP, dGTP, dTTP, and dCTP; and a blue dye indicator. The mixture was made up of 1 μL each of forward and reverse primers, 3 μL of genomic DNA, and nuclease-free water to reach a final volume of 30 µL. The PCR reaction was performed in a BIOER XP thermal cycler model TC-E-96G (BIOER Technology Co., Ltd., Hangzhou, China) under the cycling conditions mentioned in Table 4, followed by gel electrophoresis to identify the PCR products.

### 4.4. DNA Sequencing

PCR products were examined using the Sanger sequencing method by a local company (Biotrust laboratory, Amman, Jordan) using a genetic analyzer (SeqStudio™ Genetic Analyzer System with SmartStart Applied Biosystems™, Thermo Fisher Scientific Inc., Pittsburgh, PA, USA). The output of sequencing was analyzed by Mutation Surveyor (v5.2.0) and UniPro UGENE (50.0) software.

### 4.5. Data Analysis

The clinical–pathological data were analyzed by using the *t*-test and Fisher’s Exact test in GraphPad Prism 9 software. Each identified variant was compared against the gnomAD v4.1 database, ClinVar, COSMIC, and cBioPortal to characterize their population frequency and prior oncological annotations. In addition, in silico tools, PolyPhen-2 (http://genetics.bwh.harvard.edu/pph2/, accessed on 6 December 2025) and MutationTaster (https://www.genecascade.org/MutationTaster2021/, accessed on 6 December 2025), were used for the prediction of the damaging impact.

## 5. Conclusions

The results of this study reported the presence of mutations in the *AKT2* gene other than the common hotspot (E17K), highlighting the possible role of *AKT2* in the pathogenesis of PC. A percentage of 5.7% showed mutations in exon 3 of the *AKT2* gene. Therefore, further genetic studies covering the whole *AKT2* gene and more investigation into the downstream pathway are required for a better understanding of PC genetics.

## Figures and Tables

**Table 1:** Clinical data of the participants.

**Parameters**	**Data**
Number of samples (N)	123
Range of age (years)	39–96
Age (Mean)	71.57
PSA score (Mean)	53.21
Gleason score (Median)	8

**Table 2:** *AKT2* mutations in exon 3.

Mutation	Position	Mutation Type	Amino Acid Change	Codon Change	Recorded Genotype
**rs201145928**	c.151C>T	Missense Variant	Pro51Ser	CCC > TCC	TT
**rs1975585202**	c.97G>A	Missense Variant	Gly33Ser	GGC > AGC	GA
**rs1975586566**	c.85C>T	Synonymous Variant	Leu29	CTG > TTG	CT
**rs1347826243**	c.126C>T	Synonymous Variant	Pro42	CCC > CCT	CT
**rs1975588070**	-	Splice Acceptor Variant	-	A > G	AG
**rs2145305645**	c.175+40C>G	Intron Variant	-	-	CG

**Table 3:** A comparison between wild-type samples (W) and mutant samples (M) based on clinical and pathological characteristics (age, PSA level, and Gleason score); Chi-square.

Parameter	Category	*AKT2*/Exon3 Mutation			*p* Value
		Number	M	W	
**Age years**	<70	44	1	43	0.2331
	70–80	58	3	55	
	<80	20	3	17	
	No data	1	0	1	
**PSA Level ng/mL**	<4	15	0	15	0.1052
	4–20	34	4	30	
	>20	39	0	39	
	No data	35	3	32	
**Gleason score**	<7	22	1	21	0.1793
	=7	35	1	34	
	>7	63	4	59	
	No data	3	1	2	

**Table 4:** Primer sequence, conditions of the thermal cycler used to produce the PCR product for *AKT2* gene exon 3, and product size.

	Exon 3/*AKT2*		
**Forward 5′-3′**	TTG TGA GTC ACC GTC ACA CT		
**Reverse 5′-3′**	GTT AGC TTT ACA GTG GGC TC		
**Operation**	**Temp °C**	**Time**	**cycles**
**Initial denaturation**	95	5 min	1 cycle
**Denaturation** **Annealing** **Elongation**	95 60 72	30 s 30 s 40 s	40 cycles
**Final elongation**	72	5 min	1 cycle
**Product size**	290 bp		

## Data Availability

The original contributions presented in this study are included in the article. Further inquiries can be directed to the corresponding author.

## Abbreviations

PC: Prostate Cancer; AKT2: Protein Kinase B Beta; FFPE: Formalin-Fixed Paraffin-Embedded; PSA: Prostate-Specific Antigen; SNP: Single-Nucleotide Polymorphism; BRCA1: Breast Cancer Gene 1; BRCA2: Breast Cancer Gene 2; TP53: Tumor Protein p53; ATM: Ataxia Telangiectasia Mutated; HOX: Homeobox Genes; MSR1: Macrophage Scavenger Receptor 1; RNase L (HPC1): Ribonuclease L (Hereditary Prostate Cancer 1); ELAC2/HPC2: ElaC Ribonuclease Z 2/Hereditary Prostate Cancer 2; PKB: Protein Kinase B; PH: Pleckstrin Homology.

## References

[1] Falkenbach F., Le Q.C., Longoni M., Marmiroli A., Catanzaro C., Nicolazzini M., Polverino F., Tian Z., Goyal J.A., Schiavina R. (2025). Years of life lost in metastatic and locally advanced prostate cancer. Eur. Urol. Oncol..

[2] Bray F., Laversanne M., Sung H., Ferlay J., Siegel R.L., Soerjomataram I., Jemal A. (2024). Global cancer statistics 2022: GLOBOCAN estimates of incidence and mortality worldwide for 36 cancers in 185 countries. CA Cancer J. Clin..

[3] Ofner H., Kramer G., Shariat S.F., Hassler M.R. (2025). TP53 deficiency in the natural history of prostate cancer. Cancers.

[4] Markozannes G., Tzoulaki I., Karli D., Evangelou E., Ntzani E., Gunter M.J., Norat T., Ioannidis J.P., Tsilidis K.K. (2016). Diet, body size, physical activity and risk of prostate cancer: An umbrella review of the evidence. Eur. J. Cancer.

[5] Ni Raghallaigh H., Eeles R.J.F.C. (2022). Genetic predisposition to prostate cancer: An update. Fam. Cancer.

[6] Berenguer C.V., Pereira F., Câmara J.S., Pereira J.A. (2023). Underlying features of prostate cancer—Statistics, risk factors, and emerging methods for its diagnosis. Curr. Oncol..

[7] Bergengren O., Pekala K.R., Matsoukas K., Fainberg J., Mungovan S.F., Bratt O., Bray F., Brawley O., Luckenbaugh A.N., Mucci L. (2023). 2022 update on prostate cancer epidemiology and risk factors—A systematic review. Eur. Urol..

[8] Arenas-Gallo C., Owiredu J., Weinstein I., Lewicki P., Basourakos S.P., Vince R., Al Hussein Al Awamlh B., Schumacher F.R., Spratt D.E., Barbieri C.E. (2022). Race and prostate cancer: Genomic landscape. Nat. Rev. Urol..

[9] Cotter K., Rubin M.A. (2022). The evolving landscape of prostate cancer somatic mutations. Prostate.

[10] Ecke T.H., Schlechte H.H., Schiemenz K., Sachs M.D., Lenk S.V., Rudolph B.D., Loening S.A. (2010). TP53 gene mutations in prostate cancer progression. Anticancer research. Anticancer. Res..

[11] Al Zoubi M.S., Otoum R., Alorjani M.S., Al Bashir S., Al Trad B., Abualrja M.I., Al-Khatib S.M., Al-Batayneh K. (2020). TP53, SPOP and PIK3CA genes status in prostate cancer. Asian Pac. J. Cancer Prev..

[12] Angele S., Falconer A., Edwards S., Dörk T., Bremer M., Moullan N., Chapot B., Muir K., Houlston R., Norman A.R. (2004). ATM polymorphisms as risk factors for prostate cancer development. Br. J. Cancer.

[13] Leongamornlert D., Mahmud N., Tymrakiewicz M., Saunders E., Dadaev T., Castro E., Goh C., Govindasami K., Guy M., O’Brien L. (2012). Germline BRCA1 mutations increase prostate cancer risk. Br. J. Cancer.

[14] Junejo N.N., AlKhateeb S.S. (2020). BRCA2 gene mutation and prostate cancer risk: Comprehensive review and update. Saudi Med. J..

[15] Alasmar A.A., Al-Alami Z., Zein S., Al-Smadi A., Al Bashir S., Alorjani M.S., Al-Zoubi R.M., Al Zoubi M. (2024). Novel mutations in AKT1 gene in prostate cancer patients in Jordan. Curr. Issues Mol. Biol..

[16] Maier C., Vesovic Z., Bachmann N., Herkommer K., Braun A.K., Surowy H.M., Assum G., Paiss T., Vogel W. (2006). Germline mutations of the MSR1 gene in prostate cancer families from Germany. Hum. Mutat..

[17] Rökman A., Ikonen T., Seppälä E.H., Nupponen N., Autio V., Mononen N., Bailey-Wilson J., Trent J., Carpten J., Matikainen M.P. (2002). Germline alterations of the RNASEL gene, a candidate HPC1 gene at 1q25, in patients and families with prostate cancer. Am. J. Hum. Genet..

[18] Javed S., Langley S.E. (2014). Importance of HOX genes in normal prostate gland formation, prostate cancer development and its early detection. BJU Int..

[19] Rökman A., Ikonen T., Mononen N., Autio V., Matikainen M.P., Koivisto P.A., Tammela T.L., Kallioniemi O.P., Schleutker J. (2001). ELAC2/HPC2 involvement in hereditary and sporadic prostate cancer. Cancer Res..

[20] Sekhoacha M., Riet K., Motloung P., Gumenku L., Adegoke A., Mashele S.J.M. (2022). Prostate cancer review: Genetics, diagnosis, treatment options, and alternative approaches. Molecules.

[21] Salim A.-Z.M., Khalid A.-B., Bahaa A.-T., Mohammed A., Samir A.-B., Mohammad A.-H., Riyad M., Ismail M. (2018). Polymorphisms of 5’-UTR of rad51 gene in prostate cancer. Экoлoгическая генетика.

[22] Degan S.E., Gelman I.H. (2021). Emerging roles for AKT isoform preference in cancer progression pathways. Mol. Cancer Res..

[23] Kyung H.Y., Lauring J.J.O. (2015). Recurrent AKT mutations in human cancers: Functional consequences and effects on drug sensitivity. Oncotarget.

[24] Song M., Bode A.M., Dong Z., Lee M.H. (2019). AKT as a therapeutic target for cancer. Cancer Res..

[25] Sakamoto K., Arnolds D.E., Fujii N., Kramer H.F., Hirshman M.F., Goodyear L.J. (2006). Role of Akt2 in contraction-stimulated cell signaling and glucose uptake in skeletal muscle. Am. J. Physiol. Endocrinol. Metab..

[26] Mäkinen S., Datta N., Rangarajan S., Nguyen Y.H., Olkkonen V.M., Latva-Rasku A., Nuutila P., Laakso M., Koistinen H.A. (2023). Finnish-specific AKT2 gene variant leads to impaired insulin signalling in myotubes. J. Mol. Endocrinol..

[27] Altomare D.A., Testa J.R. (2005). Perturbations of the AKT signaling pathway in human cancer. Oncogene.

[28] Maekawa S., Takata R., Obara W. (2024). Molecular mechanisms of prostate cancer development in the precision medicine era: A comprehensive review. Cancers.

[29] Nandwa J.O., Mehmood A., Mahjabeen I., Raheem K.Y., Hamadou M., Raimi M.Z., Kayani M.A. (2024). miR-4716–3p and the target AKT2 Gene/rs2304186 SNP are associated with blood cancer pathogenesis in Pakistani population. Non-Coding RNA Res..

[30] Miao L., Wang B., Zhang J., Yin L., Pu Y.J.E.S., Research P. (2021). A functional SNP in miR-625-5p binding site of AKT2 3′ UTR is associated with noise-induced hearing loss susceptibility in the Chinese population. Environ. Sci. Pollut. Res. Int..

[31] Sun X.Q., Luo Y.Y., An L.W., Chu L., Huo L.L., Han X.Y., Zhou X.H., Ren Q., Ji L.N. (2011). Contribution of the Akt2 gene to type 2 diabetes in the Chinese Han population. Chin. Med. J..

[32] Manning A., Highland H.M., Gasser J., Sim X., Tukiainen T., Fontanillas P., Grarup N., Rivas M.A., Mahajan A., Locke A.E. (2017). A Low-Frequency Inactivating Variant Enriched in the Finnish Population Is Associated with Fasting Insulin Levels and Type 2 Diabetes Risk. Diabetes.

[33] Sasaki H., Okuda K., Kawano O., Yukiue H., Yano M., Fujii Y. (2008). AKT1 and AKT2 mutations in lung cancer in a Japanese population. Mol. Med. Rep..

[34] Mitala Y., Ssenkumba B., Birungi A., Kiconco R., Mutakooha M.M., Atwine R. (2024). A cross-sectional study of ERG expression and the relationship with clinicopathological features of Prostate cancer in Southwestern Uganda. Diagn. Pathol..

[35] Shorning B.Y., Dass M.S., Smalley M.J., Pearson H.B. (2020). The PI3K-AKT-mTOR pathway and prostate cancer: At the crossroads of AR, MAPK, and WNT signaling. Int. J. Mol. Sci..

[36] Glaviano A., Foo A.S., Lam H.Y., Yap K.C., Jacot W., Jones R.H., Eng H., Nair M.G., Makvandi P., Geoerger B. (2023). PI3K/AKT/mTOR signaling transduction pathway and targeted therapies in cancer. Mol. Cancer.

[37] Fujio Y., Mitsuuchi Y., Testa J., Walsh K. (2001). Differentiation. Activation of Akt2 Inhibits anoikis and apoptosis induced by myogenic differentiation. Cell Death Differ..

[38] Arboleda M.J., Lyons J.F., Kabbinavar F.F., Bray M.R., Snow B.E., Ayala R., Danino M., Karlan B.Y., Slamon D.J. (2003). Overexpression of AKT2/protein kinase Bβ leads to up-regulation of β1 integrins, increased invasion, and metastasis of human breast and ovarian cancer cells. Cancer Res..

[39] Linnerth-Petrik N.M., Santry L.A., Moorehead R., Juecker M., Wootton S.K., Petrik J. (2016). Akt isoform specific effects in ovarian cancer progression. Oncotarget.

[40] Cheng J.Q., Ruggeri B., Klein W.M., Sonoda G., Altomare D.A., Watson D.K., Testa J.R. (1996). Amplification of AKT2 in human pancreatic cells and inhibition of AKT2 expression and tumorigenicity by antisense RNA. Proc. Natl. Acad. Sci. USA.

[41] Hassan D., Menges C.W., Testa J.R., Bellacosa A. (2024). Research CC. AKT kinases as therapeutic targets. J. Exp. Clin. Cancer Res..

[42] Chau N.-M., Ashcroft M. (2003). Akt2: A role in breast cancer metastasis. Breast Cancer Res..

[43] Attoub S., Arafat K., Kamel Hammadi N., Mester J., Gaben A.-M. (2015). Akt2 knock-down reveals its contribution to human lung cancer cell proliferation, growth, motility, invasion and endothelial cell tube formation. Sci. Rep..

[44] Liu T., Zhu J., Du W., Ning W., Zhang Y., Zeng Y., Liu Z., Huang J.-A. (2020). AKT2 drives cancer progression and is negatively modulated by miR-124 in human lung adenocarcinoma. Respir. Res..

[45] Rychahou P.G., Kang J., Gulhati P., Doan H.Q., Chen L.A., Xiao S.Y., Chung D.H., Evers B.M. (2008). Akt2 overexpression plays a critical role in the establishment of colorectal cancer metastasis. Proc. Natl. Acad. Sci. USA.

[46] Zheng B., Geng L., Zeng L., Liu F., Huang Q. (2018). AKT2 contributes to increase ovarian cancer cell migration and invasion through the AKT2-PKM2-STAT3/NF-κB axis. Cell. Signal..

[47] Altomare D.A., Tanno S., De Rienzo A., Klein-Szanto A.J., Tanno S., Skele K.L., Hoffman J.P., Testa J.R. (2002). Frequent activation of AKT2 kinase in human pancreatic carcinomas. J. Cell. Biochem..

